# A Multifunctional Composite Framework with Self-Healing and Guided Wave-Based States Awareness

**DOI:** 10.3390/s26154777

**Published:** 2026-07-27

**Authors:** Shilei Wang, Yihang Cai, Qidi Shan, Lei Qiu

**Affiliations:** Research Center of Structural Health Monitoring and Prognosis, State Key Laboratory of Mechanics and Control for Aerospace Structures, Nanjing University of Aeronautics and Astronautics, Nanjing 210016, China; wangshilei1993@nuaa.edu.cn (S.W.); caiyihang@nuaa.edu.cn (Y.C.); shanqidi@nuaa.edu.cn (Q.S.)

**Keywords:** multifunctional composites structure, structural health monitoring, guided wave, state monitoring

## Abstract

Modern aeronautical engineering increasingly demands multifunctional materials that provide capabilities beyond passive load-bearing. While self-healing composites offer autonomous repair against impact damage, their practical application requires reliable in situ perception of structural states. Addressing this, this study proposes a novel multifunctional composite structural framework that synergistically integrates damage self-healing with guided wave-based structural health monitoring. Rather than treating monitoring and repair as isolated processes, this integrated strategy endows the composite with self-aware and self-healing characteristics, enabling closed-loop tracking of the dynamic “healthy–damaged–healed” processing. The interactive effects of simulated damage and various structural impact damaged states on distinct guided wave modes were systematically analyzed. Furthermore, a state index method is introduced to characterize damage propagation and accurately distinguish between evolving structural states. Experimental validations demonstrated spatial awareness: the framework achieved a minimum localization error of 5 mm for simulated damage. Crucially, for barely visible impact damage, the damaged and self-healing states were precisely localized with errors of 3 mm and 5 mm, respectively, with all overall localization errors strictly bounded below 9 mm. These findings validate the efficacy of the proposed multifunctional strategy, providing a highly reliable paradigm for the next generation of intelligent, damage-resilient aeronautical composite structures.

## 1. Introduction

In modern aerospace engineering, the design philosophy of structural components is rapidly evolving from passive load-bearing elements to intelligent, multifunctional systems. While composite materials are widely used due to their high specific strength, high specific modulus, and excellent mechanical properties [1,2], the proportion of composite materials used in aircraft structures has become an important indicator of advanced aircraft design and manufacturing capability [3]. However, during manufacturing and service processes, composite plates inevitably develop various forms of defects, especially barely visible impact damage. Such damages significantly degrade the mechanical strength and service life of the structure. Because they are challenging to detect visually, failure to locate and identify these damages in a timely manner can lead to catastrophic consequences during aircraft operation [4,5]. Consequently, endowing aircraft structures with the ability to perceive and manage damage autonomously has become a critical frontier in aerospace engineering.

As for the pronounced anisotropy of composite structures and the complex mechanisms of impact-induced damage, damage monitoring and detection in aircraft composite structures remain challenging. To ensure the safety and reliability of aircraft during service, various non-destructive testing techniques have been developed, including thermography, X-ray inspection, eddy current testing, vibration-based methods, and ultrasonic C-scan techniques [6,7,8,9,10]. Although these techniques have achieved significant progress in damage characterization, they are generally time-consuming, costly, and dependent on specialized equipment and trained operators, which may restrict their broader application for in situ and large-area monitoring in aerospace structures. Structural health monitoring (SHM) technology provides a promising alternative for real-time or in situ damage detection and condition assessment of composite structures. Various SHM techniques have been investigated, including fiber Bragg grating sensors, electromechanical impedance methods, acoustic emission techniques, and guided wave (GW)-based methods [11,12,13,14].

Owing to its high sensitivity to small-scale damage and its capability for regional monitoring, GW-based structural health monitoring has been regarded as a promising approach for damage detection and condition assessment in composite structures. When damage occurs in a structure, the GW response may undergo scattering, reflection, attenuation, and other propagation changes. As a result, signal features such as amplitude, phase, and energy may vary significantly when the monitoring signal acquired in the damaged state is compared with the baseline signal acquired in the healthy state. Therefore, structural state changes can be monitored by extracting and analyzing these signal feature variations [15,16,17]. Researchers have investigated the relationship between structural damage and GW propagation through theoretical analysis, numerical simulation, and experimental validation. In particular, the finite element method has been widely used to analyze GW propagation characteristics in composite plates and to evaluate the influence of different damage forms, such as delamination and impact damage, on GW signals with different modes [18,19]. Zheng et al. investigated the interaction between GW and structural features or damage in composite stiffened panels, including stiffener-skin debonding and low-velocity impact damage in the skin [20]. In addition, an impedance- and Lamb-wave-based SHM method was developed for cryotank composite structures by integrating environmental awareness, damage prewarning, sensor self-diagnosis, and damage detection. This method was validated through cryogenic delamination monitoring tests, demonstrating its effectiveness for composite structures under low-temperature conditions [21]. These studies collectively demonstrate the potential of GW-based methods for damage monitoring in different composite structural components.

When damage occurs in a structure, the GW response may exhibit scattering, reflection, attenuation, and other propagation changes. Structural state information can be obtained by extracting characteristic differences between the baseline signal acquired in the healthy state and the monitoring signal acquired in the damaged state [22,23]. The damage index is an effective metric for quantifying these signal variations and characterizing changes in structural states [24,25]. Therefore, if a direct relationship can be established between the extracted signal feature variations and known damage parameters, these features can be used as indicators for damage characterization and assessment.

After identifying signal variations associated with structural damage, imaging-based methods can be further employed to localize the damage position. GW-based imaging algorithms provide an effective approach for detecting structural defects over relatively large monitoring areas while maintaining sensitivity to small-scale damage [26,27]. In recent years, damage imaging methods based on piezoelectric transducer (PZT) arrays and GW have attracted increasing attention in the monitoring of complex aerospace structures. Representative methods include delay-and-sum imaging, probability-based diagnostic imaging, time-reversal imaging, and phased-array imaging [28,29,30,31]. Among these methods, delay-and-sum imaging has been widely used because of its simple implementation, suitability for large-area monitoring, and capability to localize different types of damage. Qiu et al. applied this method to realize imaging localization of multiple damage sites in composite structures with bolt holes and stiffeners [32]. Xu et al. proposed a high-resolution Lamb wave inspection method combining sparse reconstruction with delay-and-sum imaging, and validated it on a quasi-isotropic laminated CFRP plate with simulated damage [33]. Yue et al. compared delay-and-sum, signal-difference, RAPID, and Voronoi-based algorithms for damage localization in CFRP structures [34].

While SHM can serve as a “nervous system” for damage perception, conventional composite structures still lack an intrinsic “immune system” for damage recovery. Once damage is detected but not repaired in time, structural performance may continue to deteriorate, leading to reduced load-bearing capacity and service reliability. Conventional manual repair methods, such as mechanical fastening and adhesive bonding [35], are generally difficult to implement during flight operations and are often ineffective for repairing internal micro-damage. Moreover, these methods may require drilling or the introduction of additional repair materials, resulting in weight increase and potential secondary damage to composite fibers. To overcome these limitations, self-healing composites have been developed to provide intrinsic damage repair capability through mechanisms such as hollow fibers, microcapsules, reversible chemical reactions, and shape memory effects [36,37]. Among these approaches, microcapsule-based self-healing technology has attracted considerable research attention. This approach involves encapsulating a healing agent within a protective shell to form microcapsules, which are subsequently incorporated into the composite matrix. When microcracks develop in the structure, the embedded microcapsules can rupture locally under crack-induced stress, releasing the healing agent into the damaged region. The released healing agent then fills the local defects and cures or solidifies under appropriate environmental or triggering conditions, thereby enabling the repair of microcracks or delamination damage. Microcapsule-based self-healing technology has been extensively investigated since White et al. first introduced capsule-based self-healing into polymeric materials [38]. Kessler et al. incorporated self-healing materials into braided composite structures and experimentally verified the recovery of mode-I interlaminar fracture toughness [39]. For impact damage in composite materials, Yin et al. investigated the self-healing performance of composite structures subjected to low-energy impact loading [40]. Patel et al. further evaluated the repair capability under low-velocity impact and compression-after-impact conditions, demonstrating that impact damage could be partially repaired when the healing agent sufficiently filled the damaged volume [41]. Extensive efforts have also been devoted to improving self-healing technologies by developing novel healing materials and optimizing the healing agents encapsulated within microcapsules.

Although self-healing composites provide a promising solution for damage repair, their internal healing systems and state evolution processes are more complex than those of conventional composite structures. In conventional GW-based SHM, the monitoring objective is generally to identify the difference between the healthy baseline and the damaged state, namely a binary healthy-damaged evaluation. However, for self-healing composite structures, impact damage is followed by the release, flow, and curing of healing agents within the damaged region. As a result, the structure does not simply remain in a damaged state, but evolves into a new self-healing state that is different from both the original healthy state and the impact-damaged state. This multi-state evolution introduces additional changes in local stiffness, interfacial continuity, and GW propagation characteristics, which cannot be fully described by conventional binary damage monitoring.

To address this issue, this study proposes a multifunctional composite structural framework that integrates self-healing capability with GW-based state awareness. The main novelty of this work is the extension of conventional guided-wave SHM from binary healthy-damaged monitoring to multi-state evolutionary monitoring involving healthy, damaged, and self-healing states. Specifically, the effects of different structural states on different GW modes are systematically investigated, and a state index is established to characterize the structural evolution induced by damage and self-healing. Furthermore, a GW imaging algorithm is employed to realize spatial localization of both the initial impact damage and the subsequent self-healing region. Therefore, the proposed framework enables simultaneous state identification and spatial awareness of self-healing composite structures, providing a promising strategy for the development of next-generation intelligent and damage-resilient aeronautical structures.

## 2. The Basic Principle of the Multifunctional Composite Structural Framework

Unlike conventional composite structures, self-healing composite structures contain pre-embedded healing agents that can be released when impact-induced cracks or delaminations rupture the core–shell healing system. For thermoset composites, the released healing agents do not diffuse through the intact matrix; instead, they flow into the damaged region through damage-induced pathways, such as matrix cracks, delamination gaps, and local interfacial debonding regions. Driven by capillary action and assisted by the prescribed heating condition, the low-viscosity healing agents fill local defects and subsequently cure or solidify, thereby enabling repair of local delamination, matrix cracking, and microcrack. Therefore, after impact loading, such structures do not simply undergo a unidirectional degradation process from a healthy state to a damaged state, but instead exhibit a multi-stage state evolution process involving the healthy, damaged, and self-healing states.

**Figure 1:**
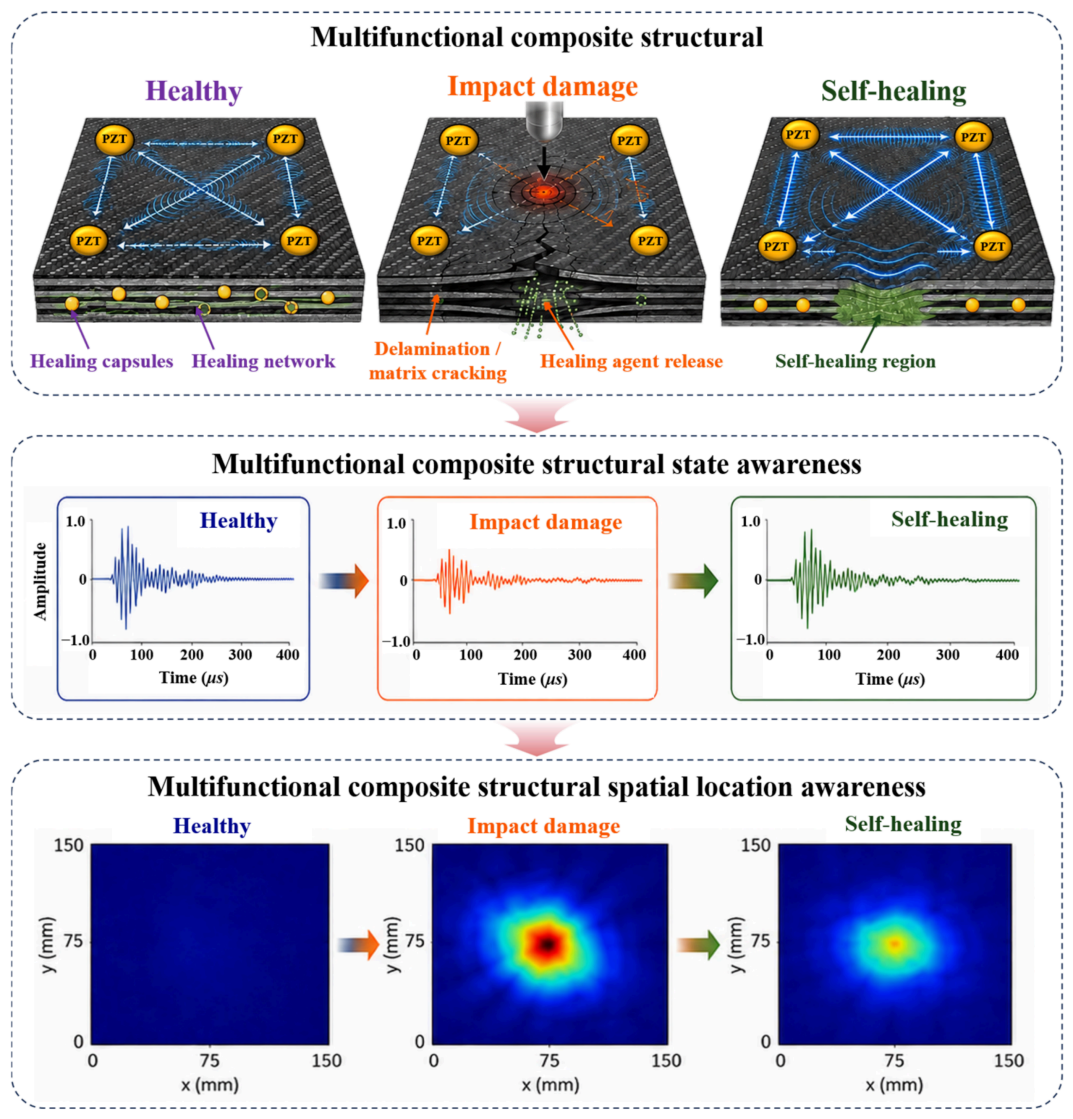
The basic principle of the multifunctional composite structural framework.

To provide the necessary self-awareness for this dynamic process, a piezoelectric GW network is integrated as the “nervous system” for structure. The unique self-healing mechanism induces dynamic variations in local stiffness, interfacial bonding conditions, material continuity, and corresponding GW propagation paths. Based on this physical mechanism, this study leverages multidimensional GW responses to track structural evolution. By comparatively analyzing the signal characteristics across the three distinct states, characteristic features such as amplitude attenuation, phase shift, and energy distribution are extracted to effectively distinguish different service states of the structure. Furthermore, by coupling the piezoelectric sensor array with advanced GW imaging algorithms, the framework achieves spatial awareness, enabling accurate in situ localization of both the initial impact damage and the subsequent healing region. The basic principle of the multifunctional composite structural framework is shown in Figure 1.

The key research of this study lies in extending GW monitoring from the conventional binary identification of healthy and damaged states in composite structures to the multi-state evolutionary monitoring of healthy, damaged, and self-healing states in multifunctional intelligent structures. The proposed framework can not only monitor the occurrence of impact damage, but also capture the recovery and reconstruction characteristics of GW propagation induced by the self-healing states. This provides a new technical route for damage diagnosis, healing-effect evaluation, and service reliability monitoring of multifunctional composite structures.

## 3. Compositional Architecture of Multifunctional Composite Structures

### 3.1. The Self-Healing Carbon Fiber Composite Specimen

**Figure 2:**
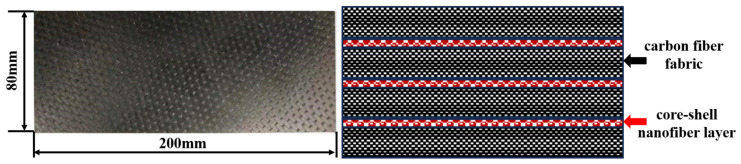
The size and internal components of self-healing composite structure.

The self-healing composite structure was used as the experimental specimen. The laminate consisted of 28 plies with a stacking sequence of (45/0/-45/0/0/90/0)_2s_, and its dimensions were 200 mm × 80 mm × 2.8 mm (length × width × thickness). The specimen was fabricated using T800 unidirectional carbon fiber fabric and an embedded core–shell nanofiber-based self-healing system. The core–shell nanofibers contained a self-healing epoxy resin system and a corresponding curing agent. The shell material was polyacrylonitrile (PAN), which was used to encapsulate the low-viscosity healing agent. The core–shell nanofiber layer, with an average thickness of approximately 37 μm, was positioned between adjacent carbon fiber layers, as shown in Figure 2.

When impact-induced cracks, delamination, or local interfacial damage occur in the composite structure, the PAN shell of the core–shell nanofibers can rupture locally, allowing the encapsulated healing agent to be released. For this thermoset composite, the released healing agent does not diffuse through the intact matrix; instead, it flows into the damaged region through damage-induced pathways, such as matrix cracks, delamination gaps, and local interfacial debonding regions. Under the prescribed thermal activation condition of 150 °C for 30 min, the healing agent cures or solidifies within the damaged region, thereby contributing to the repair of local microcracks and delamination damage.

### 3.2. Integration of PZT Sensors with Structures

**Figure 3:**
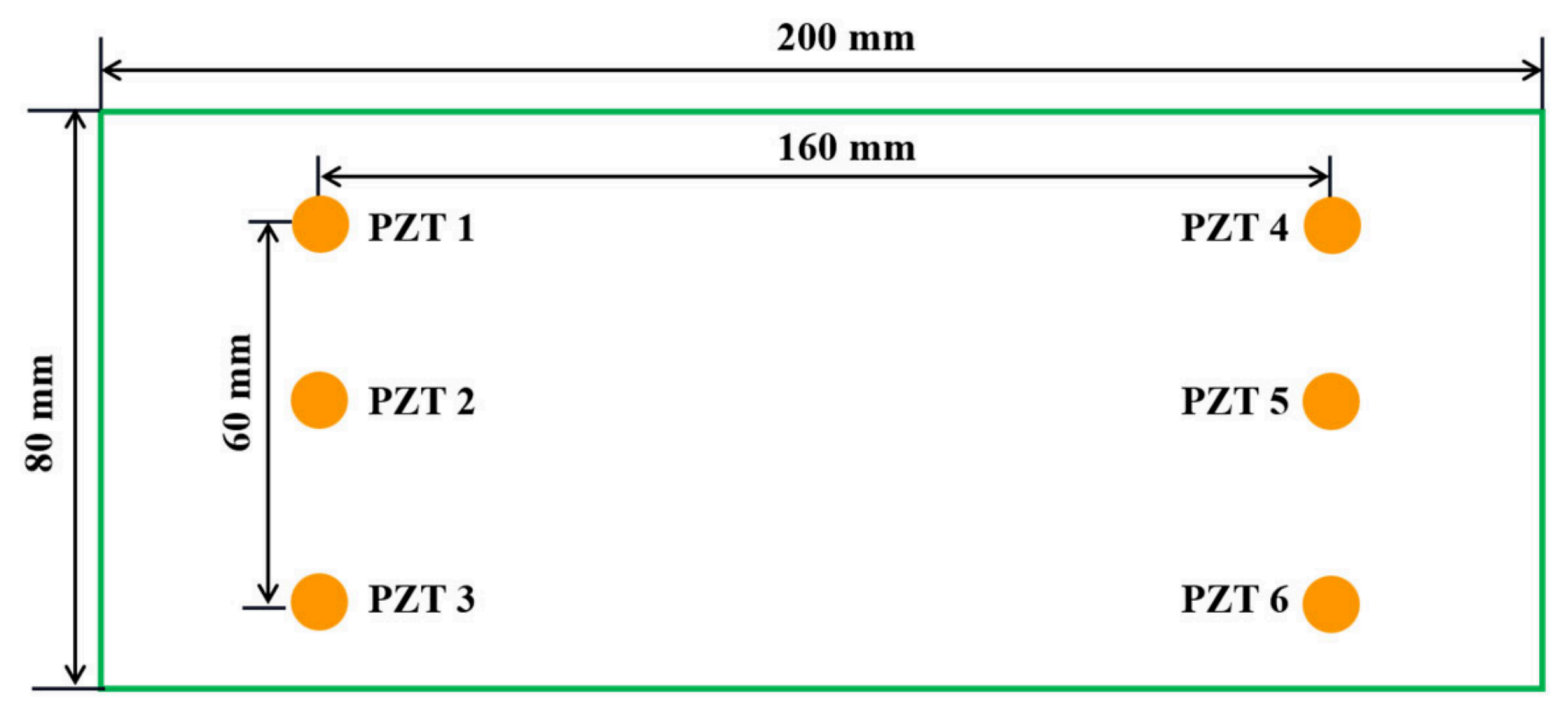
The sensor layout of self-healing composite material structure.

A piezoelectric sensor array consisting of six zirconate titanate ceramic transducers was surface-bonded to the composite structure using epoxy adhesive. The sensors were numbered from PZT 1 to PZT 6, and the center-to-center spacings between adjacent PZTs in the longitudinal and transverse directions were 160 mm and 30 mm, respectively, as shown in Figure 3.

### 3.3. Definition of Damage in Multifunctional Composite Structures

During the experiment, two distinct forms of damage were applied: one consisting of simulated damage of varying dimensions, and the other comprising impact damage of different energy levels.

The simulated damages: The sealant was chosen as the simulated damage on the surface of the structure, to study the propagation mechanism of GW on structures and the effects of damage on signal characteristics. The thickness of the damage was 1 mm, with areas of 3 mm × 3 mm, 5 mm × 5 mm, 7 mm × 7 mm, 9 mm × 9 mm, and 15 mm × 15 mm, respectively. And these damages were marked as Level 1, Level 2, Level 3, Level 4 and Level 5.

**Figure 4:**
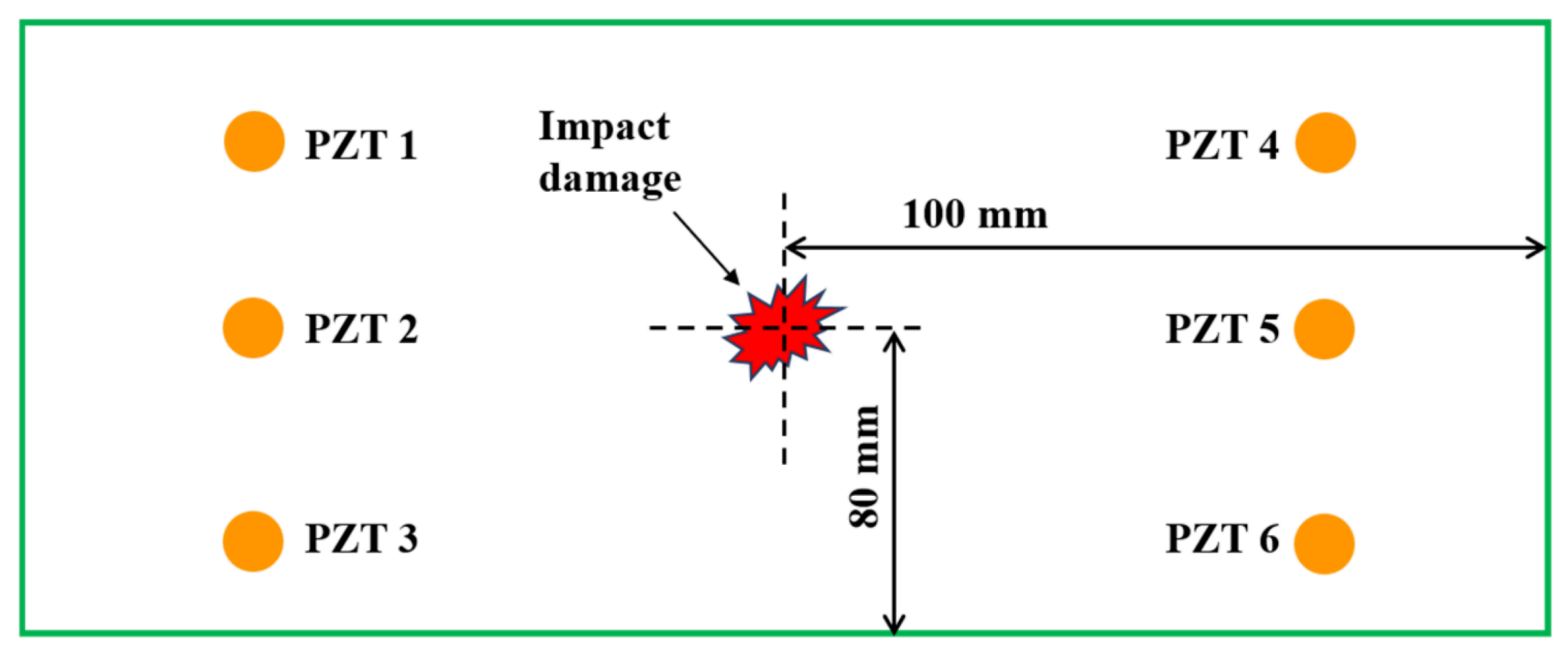
The location of impact damage.

The impact damages: The impact loading of different energy levels was applied to the specimen using a drop-weight impact loader. The impact energy was obtained through the free fall of the impact hammer at various heights. The impact hammerhead was hemispherical with a diameter of 12 mm, effectively serving as an impact source with a mass of 1 kg. The self-healing composite panel was secured in the fixture to achieve complete fixation during the experiment. The impact energy could be adjusted to 2 J, 4 J, 6 J, 8 J and 10 J respectively by controlling the drop height of the hammer; the location of impact damage is shown as follows in Figure 4.

### 3.4. Acquisition of Signals in Different States

The PZTs were connected to an integrated SHM system [42], which was used to control all six PZTs to actuate and sense GW signals under different states of the composite panel. The pitch-catch node was used, and the sensor channel was defined as PZT*_i_*–PZT*_j_*. The excitation signal adopted in this validation was a three-cycle sine burst modulated by the Hanning window with a central frequency of 70 kHz and 160 kHz. These two frequencies were selected based on preliminary signal observations to obtain recognizable GW packets with sufficient amplitudes and clear modal characteristics. Specifically, the 70 kHz excitation provided a distinct A0 mode response, whereas the 160 kHz excitation provided a relatively clear S0 mode response. In this study, the A0 mode refers to the fundamental antisymmetric mode, which is mainly associated with flexural and out-of-plane motion, while the S0 mode refers to the fundamental symmetric mode, which is mainly associated with extensional and in-plane motion [43].

During the experiment, GW signals were acquired when the structure was observed at states of healthy, damaged and self-healing respectively.

1. The structure was healthy. The baseline GW signal of all the channels was acquired under room temperature.
2. The structure was damaged. When the structure was in different levels of simulated damages and impact damages, it was considered as the damaged states of the structure. As with the healthy state, data acquisition takes place in the same way.
3. After impact damage, the specimen was placed in a thermostatic oven at 150 °C for 30 min to provide the required thermal activation condition for the self-healing process. After cooling to room temperature, GW signals were acquired using the same experimental procedure as that used for the healthy and damaged states.

## 4. Methods and Strategies for Multifunctional Composite Structural State Awareness

### 4.1. The Method for Structural States Awareness

The state index (SI) was calculated to characterize the influence of damage or self-healing on the GW signal characteristics. Physically, the state index represents the normalized waveform discrepancy between the original healthy baseline signal and the monitoring signal acquired in the damaged or self-healing state. Therefore, it does not reflect only a single signal feature, but rather the combined effect of multiple GW variations induced by structural state changes, including amplitude attenuation, phase shift, waveform distortion, and energy redistribution. In this study, the state index was calculated for selected sensing channels at representative excitation frequencies, namely 70 kHz for the A0 mode and 160 kHz for the S0 mode, as shown in Equation (1).

(1) $$SI=\sqrt{\frac{\int_{t_{1}}^{t_{2}}{\left[H\left(t\right)-M\left(t\right)\right]}^{2}dt}{\int_{t_{1}}^{t_{2}}H^{2}\left(t\right)dt}}$$

Among them, *H*(*t*) denotes the baseline signal acquired in the healthy state, while *M*(*t*) denotes the monitoring signal acquired in the damaged or self-healing state. *t*_1_ and *t*_2_ are the starting and ending points of the signal in the time domain. It should be noted that the original healthy baseline signal was used as the common reference for both the damaged state and the self-healing state. Therefore, the state index of the self-healing state represents the deviation of the self-healing state response from the original healthy state response. This unified baseline reference enables direct comparison of the healthy, damaged, and self-healing states.

### 4.2. The Method for Structural Spatial Location Awareness

**Figure 5:**
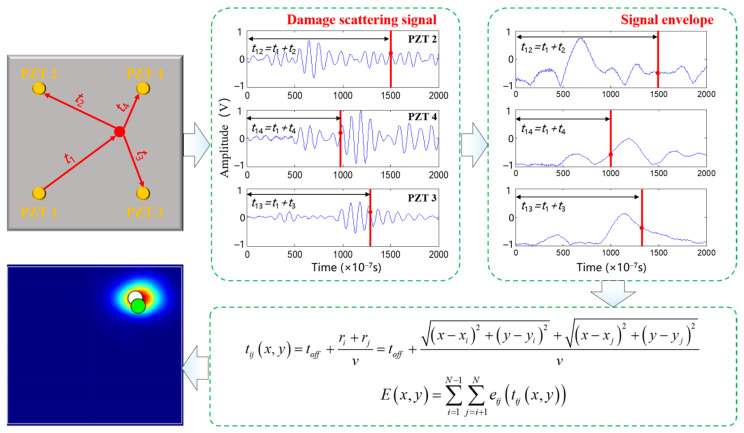
The process of damaged or self-healing imaging localization.

The localization of damage and self-healing regions in the structure was investigated using the GW array imaging algorithm. As shown in Figure 5, in this process, each pixel (*x*, *y*) in the monitoring region is regarded as a potential damage or self-healing point. For a given actuator–sensor path, the theoretical arrival time *t_ij_* (*x*, *y*) represents the propagation time of the scattering signal from the actuator to the pixel and then from the pixel to the sensor. The envelope amplitude *e_ij_* (*t_ij_* (*x*, *y*)) at this arrival time is extracted as the contribution of this sensing path to the current pixel. By summing the envelope-amplitude contributions from all actuator–sensor paths, the imaging intensity *E*(*x*, *y*) of each pixel is obtained. Therefore, a larger imaging intensity indicates a higher probability that the damage or self-healing region is located at this pixel. The specific implementation steps are as follows.

The entire monitoring area was discretized into a two-dimensional pixel grid. The position of each pixel was represented by its coordinate (*x_m_*, *y_m_*), where *m* and *n* denote the pixel indices in the *x*- and *y*-directions, respectively. The pixel coordinates were calculated in Equation (2).

(2) $$\left\{\begin{array}{c} x_{m}=x_{\min}+\left(m-1\right)\times \frac{x_{\max}-x_{\min}}{C_{x}-1},m=1,2,\ldots C_{x}\\ y_{n}=y_{\min}+\left(n-1\right)\times \frac{y_{\max}-y_{\min}}{C_{y}-1},n=1,2,\ldots C_{y} \end{array}\right.$$

Among them, *x_min_* and *x_max_* are the minimum and maximum values of the *x* coordinates of all sensors, and *y_min_* and *y_max_* are the minimum and maximum values of the *y* coordinates of all sensors. *C_x_* and *C_y_* denote number of pixels in the *x*- and *y*-directions, respectively. When the same number of pixels is used in both directions, *C_x_* = *C_y_* = *C*, and the monitoring region is discretized into a *C* × *C* grid.

For each actuator–sensor path, the baseline signal acquired in the healthy state was compared with the monitoring signal acquired in the damaged or self-healing state to obtain the corresponding scattering signal. The signal envelope was then extracted for each sensing path.

The estimated arrival time of the scattering signal for each pixel on each channel, denoted as *t_ij_*(*x*, *y*), was calculated, as shown in Equation (3).

(3) $$t_{ij}\left(x,y\right)=t_{off}+\frac{r_{i}+r_{j}}{v}=t_{off}+\frac{\sqrt{{\left(x-x_{i}\right)}^{2}+{\left(y-y_{i}\right)}^{2}}+\sqrt{{\left(x-x_{j}\right)}^{2}+{\left(y-y_{j}\right)}^{2}}}{v}$$

Among them, *v* is the mean value of velocity for all channels, *t_off_* is the time offset corresponding to the excitation signal, and (*x_i_*, *y_i_*) and (*x_j_*, *y_j_*) are the coordinates of excitation element and sensing element for the channel.

Calculate the pixel value *E*(*x*, *y*) for each point in the monitoring area and synthesize the image, as shown in Equation (4).

(4) $$E\left(x,y\right)=\sum_{i=1}^{N-1}\sum_{j=i+1}^{N}e_{ij}\left(t_{ij}\left(x,y\right)\right)$$

Among them, *e_ij_* (*t_ij_* (*x*, *y*)) is the corresponding value of the point in the damaged or self-healing scattering signal envelope *e_ij_* of each channel, and *N* is the total number of channels within the monitoring area.

The localization error is calculated by recording the actual center position of the damaged or self-healing as (*x_act_*, *y_act_*), the center of localization is obtained from the imaging result as (*x_loc_*, *y_loc_*), and the localization error is calculated by Equation (5).

(5) $$\varepsilon_{loc}=\sqrt{{\left(x_{act}-x_{loc}\right)}^{2}+{\left(y_{act}-y_{loc}\right)}^{2}}$$

Additionally, the error is evaluated by calculating the positioning error as a percentage of the maximum distance *d*_*adj*,*max*_ between adjacent sensors, as shown in Equation (6).

(6) ηloc=εlocdadj,max

In this paper, the value of *d_adj_*_,*max*_ was 160 mm.

## 5. Results and Discussion for Multifunctional Composite Structural State Awareness

### 5.1. The Structural State Awareness Based on Signal Characteristics and State Index

#### 5.1.1. Signal Characteristics Under Simulated Damage

In the process of signal processing, different dominant signal modes may exist at different excitation frequencies. A0 mode and S0 mode may exhibit different sensitivities to damage because of their different displacement fields and propagation characteristics. Therefore, it is important to select the dominant mode with large amplitude and clear separation from other modes at a specific frequency. Based on the measured signal characteristics, the A0 mode was selected as the dominant mode at 70 kHz, while the S0 mode was selected as the dominant mode at 160 kHz.

**Figure 6:**
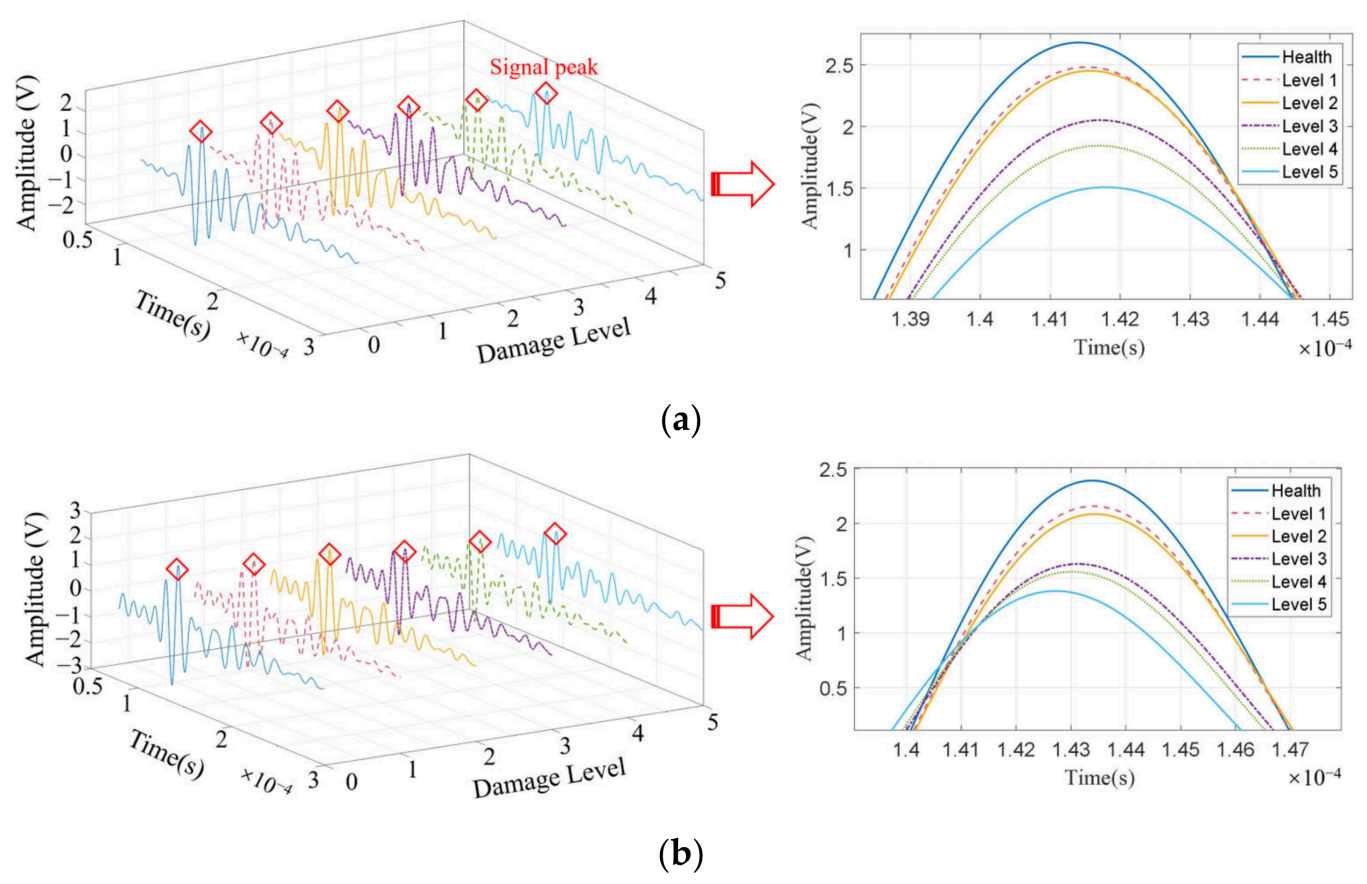
A0 mode GW signals under different levels of simulated damage at 70 kHz: (**a**) PZT_1_–PZT_5_ and (**b**) PZT_3_–PZT_5_.

The A0 mode of PZT_1_–PZT_5_ and PZT_3_–PZT_5_ were selected for analysis when different levels of simulated damages. As shown in Figure 6, the distance between the two channels and the distance between them and the damage were the same, and the signal amplitude was approximately the same in the healthy state. Additionally, it could be observed that the signal amplitude of PZT_1_–PZT_5_ exhibited a significant downward trend as the degree of damage increased, with the maximum amplitude decreasing by approximately 41.5%. A similar pattern was observed for PZT_3_–PZT_5_, with the maximum amplitude decreasing by about 41.6%. This demonstrated that simulated damages on the multifunctional composite structure significantly affect the propagation of the A0 mode.

**Figure 7:**
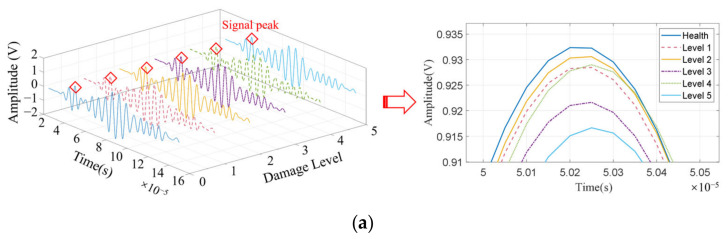

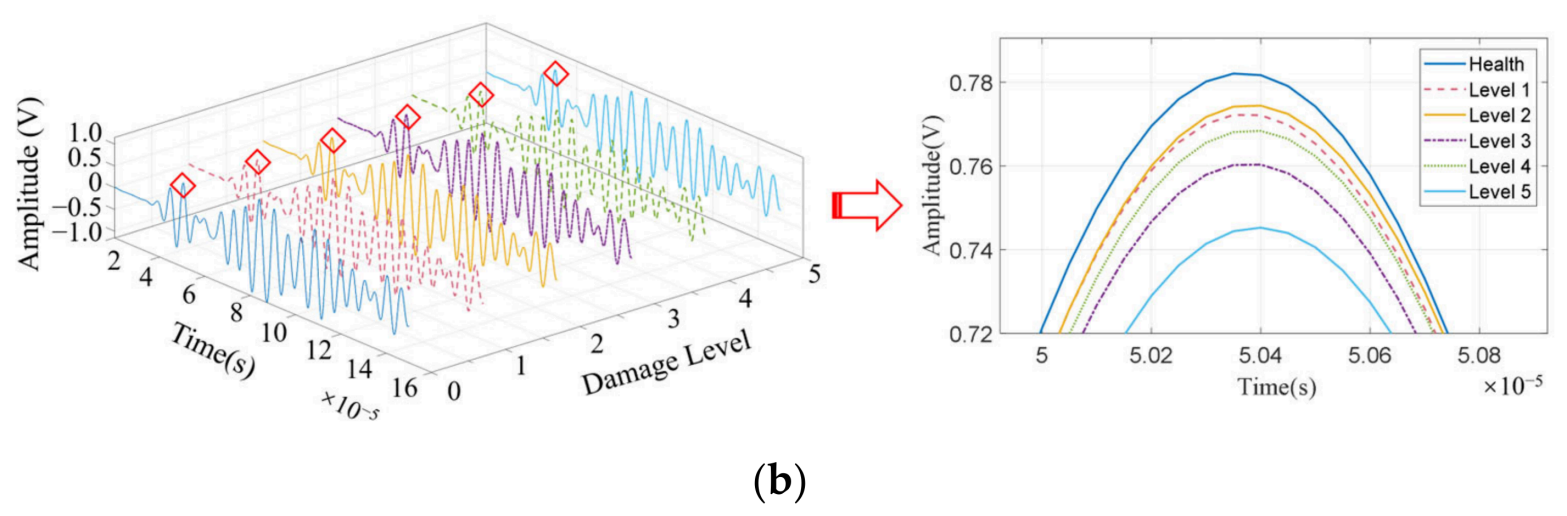
S0 mode GW signals under different levels of simulated damage at 160 kHz: (**a**) PZT_1_–PZT_5_ and (**b**) PZT_3_–PZT_5_.

The S0 mode of the signal acquired in different structural states was shown in Figure 7. The amplitude of the GW signal was largest when the structure was in a healthy state and smallest when the damage area was at Level 5. However, the amplitude change of the signal was about 1.7%, and the degree of change was much smaller than the A0 mode. Additionally, the signal amplitude did not gradually decrease with the increase in the damage for simulated damages of different sizes. The same mode was also observed for PZT_3_–PZT_5_.

#### 5.1.2. Signal Characteristics Under Impact Damage and Self-Healing States

We applied 2 J of impact energy to the structure to generate barely visible impact damage, before placing it in a specified self-healing environment. The signals were obtained at healthy state, damaged state, and self-healing state.

**Figure 8:**
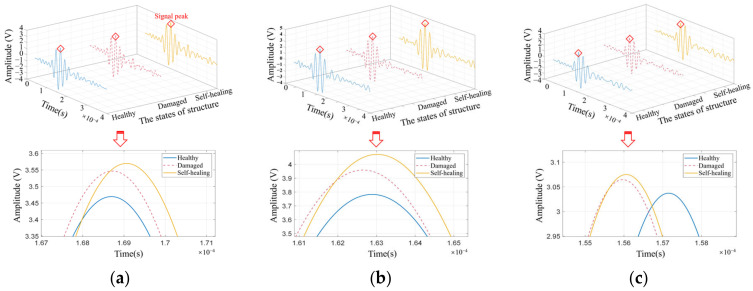
A0-mode GW signals under healthy, impact damage and self-healing states at 70 kHz: (**a**) PZT_1_–PZT_6_; (**b**) PZT_2_–PZT_5_; and (**c**) PZT_3_–PZT_4_.

The signal characteristics of the A0 mode for PZT_1_–PZT_6_, PZT_2_–PZT_5_, and PZT_3_–PZT_4_ sensing channels at 70 kHz, respectively, were analyzed, as shown in Figure 8. For each structural state, the signal was acquired three times under the same experimental conditions, and the plotted waveform represents the averaged response of the repeated measurements. There were no mode conversions in different structural states. The signal characteristics changed significantly under different structural states, and the signal amplitude in the self-healing state was greater than that in the other two states and the minimum in the healthy state. The maximum amplitude of the difference signal which was obtained by subtracting the monitoring signal from the baseline signal was about 0.4 V.

**Figure 9:**
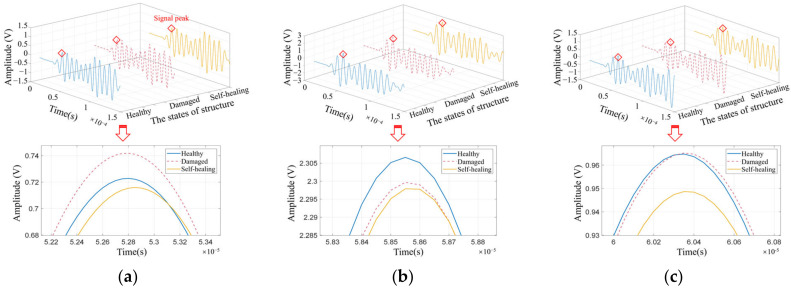
S0 mode GW signals under healthy, impact damage and self-healing states at 70 kHz: (**a**) PZT_1_–PZT_6_; (**b**) PZT_2_–PZT_5_; and (**c**) PZT_3_–PZT_4_.

The signal characteristics of the S0 mode were further analyzed for the PZT_1_–PZT_6_, PZT_2_–PZT_5_, and PZT_3_–PZT_4_ sensing channels at 160 kHz, as shown in Figure 9. No obvious mode conversion was observed among the healthy, damaged, and self-healing states. However, the relative amplitude levels of the three states were not consistent among different sensing channels. For example, the baseline signal in Figure 9a was slightly lower than the damage-state signal, whereas the baseline signal was higher in Figure 9b and comparable to the other states in Figure 9c. This difference is mainly attributed to the weak sensitivity of the S0 mode to the shallow impact-induced microcracks and local self-healing region, as well as the combined influence of the propagation path and structural anisotropy. Since the maximum difference among the S0 mode signals was only about 0.04 V, the amplitude variation was small and did not show a stable or monotonic trend among different channels. Therefore, the S0 mode was considered insufficiently sensitive for reliable state identification in this study.

#### 5.1.3. The State Index for Simulated Damage

**Figure 10:**
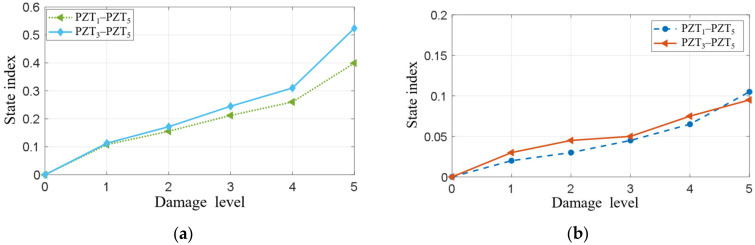
The state index of different channels with different signal modes. (**a**) A0 and (**b**) S0.

The state index of the structure under simulated damage of different sizes was calculated using the proposed method, as shown in Figure 10. It could be observed that for the A0 mode of PZT_1_–PZT_5_, there was a positive correlation between the value of the state index and the simulated damage size. PZT_3_–PZT_5_ also showed the same pattern. However, for the S0 mode, although the state index also showed a gradual upward trend, the value was relatively small. This indicated that the signal differences between different states were inconspicuous. It also proved that the monitoring ability of simulated damages using the A0 mode was better than that of the S0 mode.

#### 5.1.4. The State Index for Impact Damage and Self-Healing States

**Figure 11:**
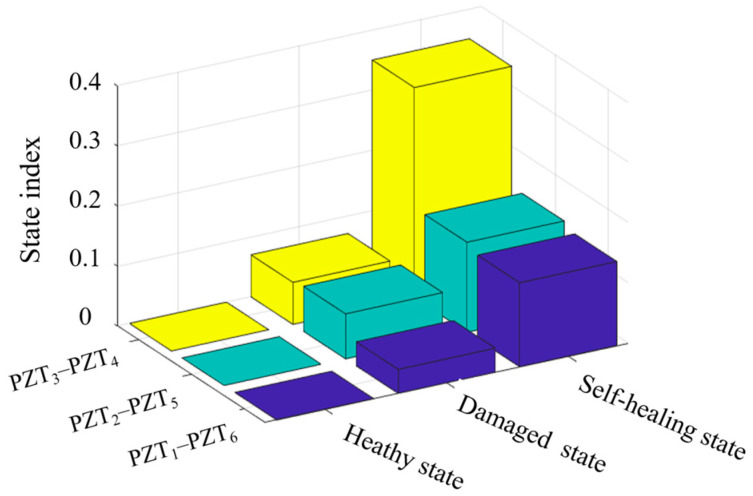
The state index of different channels for A0 mode.

For multifunctional composite structures subjected to impact damage, the state index of the A0 mode of PZT_1_–PZT_6_, PZT_2_–PZT_5_, and PZT_3_–PZT_4_ is shown in Figure 11. The values of the damaged state index and self-healing state index were calculated based on the healthy state, respectively. It could be observed that the value of the self-healing index was significantly greater than the damaged state index. Among them, the maximum value of the damaged state index was about 0.1, and the maximum value of the self-healing index was about 0.35. This meant that the state index obtained based on the proposed method can clearly distinguish between the damaged state and the self-healing state.

**Figure 12:**
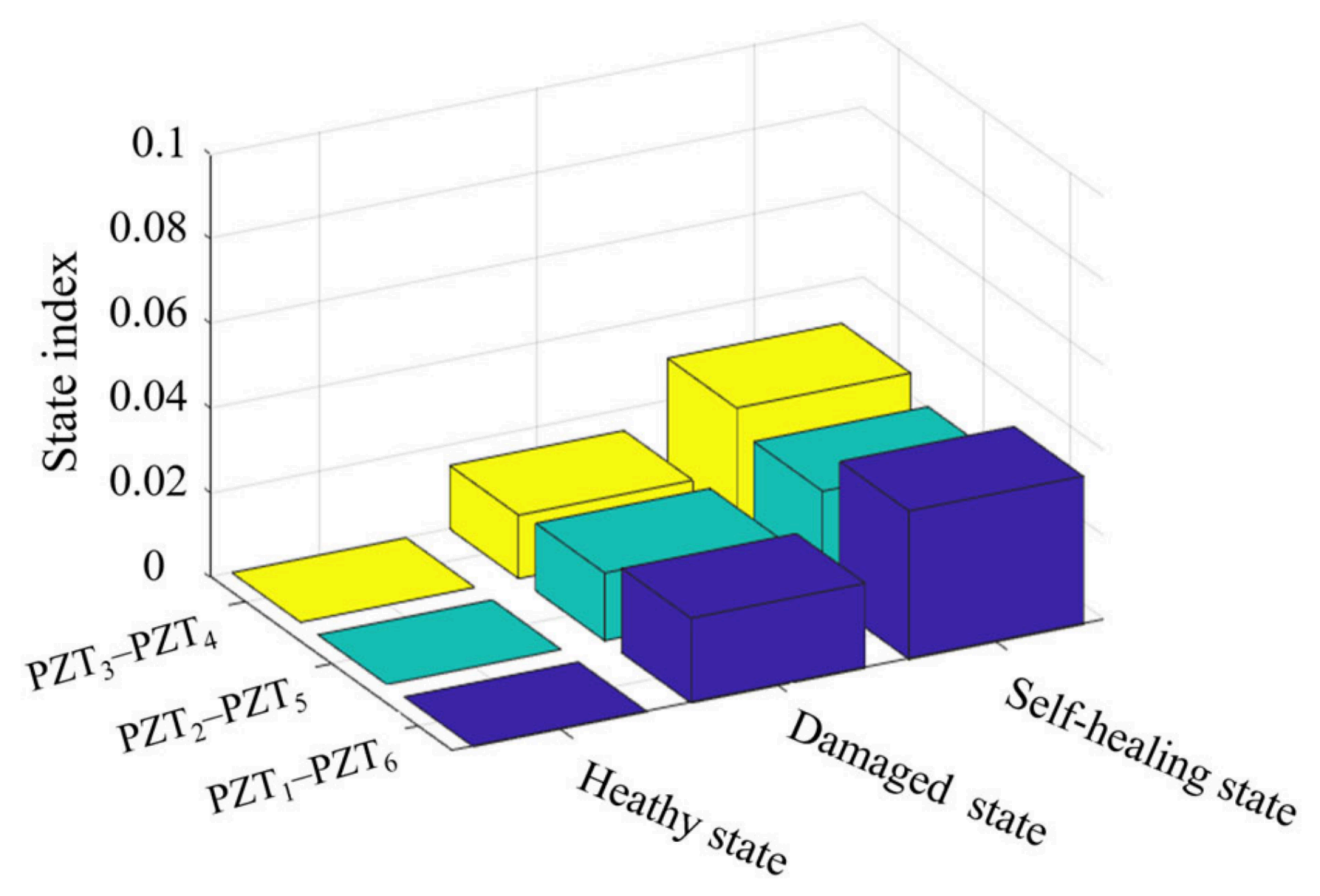
The state index of different channels for S0 mode.

As for the state index of the S0 mode of PZT_1_–PZT_6_, PZT_2_–PZT_5_, and PZT_3_–PZT_4_, it was shown in Figure 12. Although the self-healing index was still greater than the state index, the value of these indices was too small to be effective in characterizing structural state changes. Among them, the maximum value of the state index was about 0.02, and the maximum value of the self-healing index was about 0.04.

It can be observed from Figure 11 and Figure 12 that both the damaged state and the self-healing state caused variations in the GW signals and the corresponding state index. Compared with the healthy state, the state index of the self-healing state was higher than that of the damaged state, especially for the A0 mode. This indicates that the self-healing process did not simply restore the structure to its original healthy condition, but introduced a new structural state with distinguishable GW characteristics.

For the 2 J impact case, no visible dent or surface crack was observed because of the low impact energy. However, barely visible impact damage may still induce local microcracks, interfacial debonding, or shallow delamination inside the composite. When GW propagates through the impact region, these defects can cause local scattering, reflection, attenuation, and phase variation, resulting in a nonzero state index compared with the healthy baseline.

After the impact-damaged specimen was subjected to the prescribed self-healing condition of 150 °C for 30 min, the core–shell PAN nanofiber system was activated. The released healing agent flowed into the damage-induced pathways and subsequently cured or solidified in the damaged region. Although this process can repair local microcracks and improve structural continuity, the healed region contains polymerized healing material that differs from the original composite matrix. Therefore, the local stiffness, interfacial condition, and material continuity in the self-healing region are different from both the original healthy structure and the impact-damaged structure. As a result, the GW response in the self-healing state exhibits more evident changes relative to the healthy baseline, leading to a higher state index than that in the damaged state.

The comparison between the A0 and S0 modes further shows that the A0 mode is more suitable for identifying the structural state evolution in this study. The state index values of the S0 mode were relatively small, and the signal variations among the healthy, damaged, and self-healing states were not sufficiently distinct. In contrast, the A0 mode showed more pronounced and consistent changes. This is mainly because the S0 mode is dominated by in-plane motion, whereas the A0 mode is dominated by flexural and out-of-plane motion. Since the low-energy impact mainly caused shallow microcracks, local interfacial changes, and out-of-plane disturbance, the A0 mode was more sensitive to the damage and self-healing effects. Therefore, the A0 mode signal features and the corresponding SI provide a more effective basis for distinguishing the healthy, damaged, and self-healing states of the multifunctional composite structure.

### 5.2. The Structural Spatial Location Awareness

#### 5.2.1. Location Awareness for Simulated Damage

**Figure 13:**
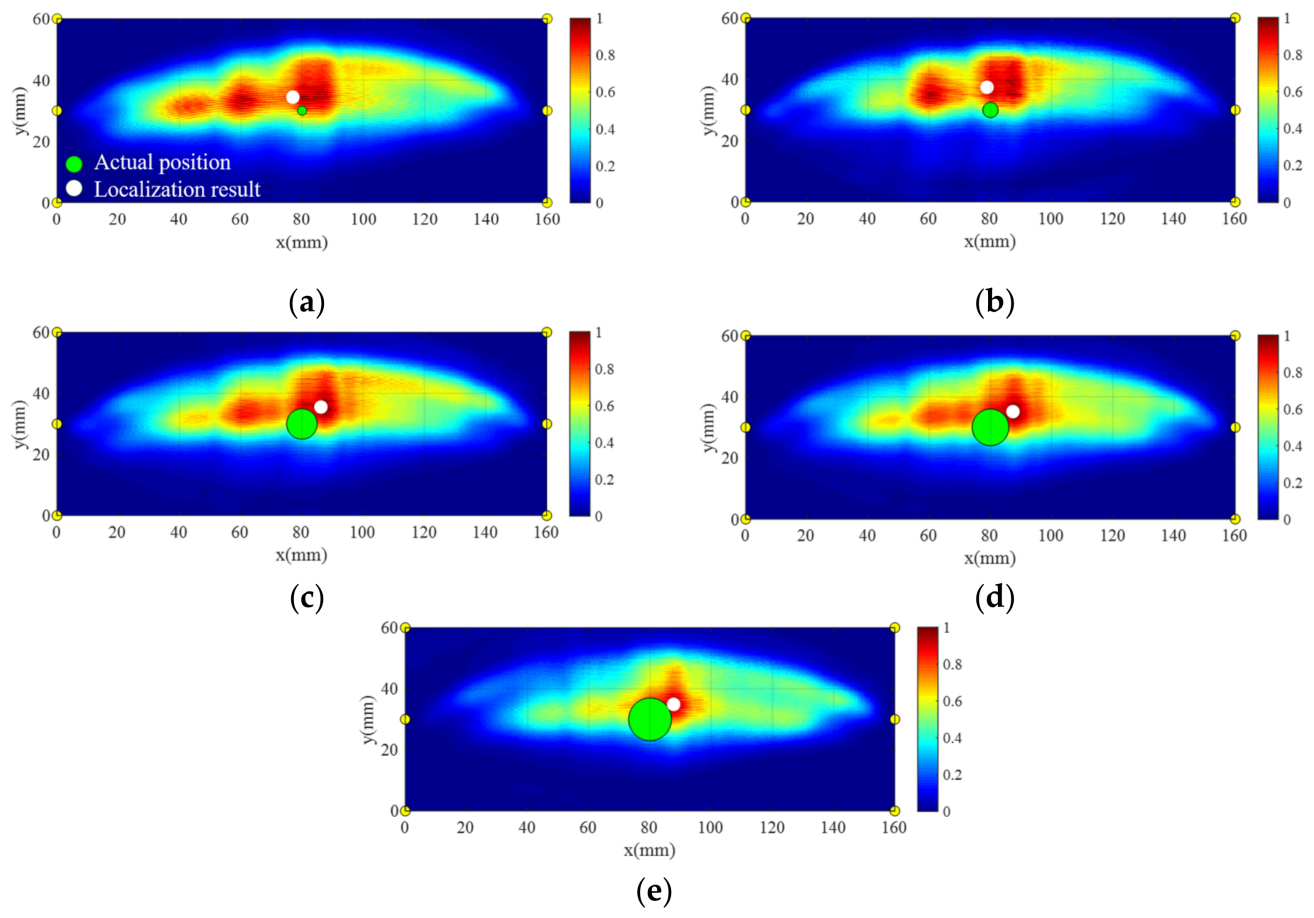
Localization results of simulated damages on multifunctional composite structures. (**a**) Level 1; (**b**) Level 2; (**c**) Level 3; (**d**) Level 4 and (**e**) Level 5.

The imaging localization results of simulated damages on multifunctional composite structures with different areas using the proposed method are shown in Figure 13. All the location errors were less than 10 mm. The minimum localization error was 5.00 mm at damage Level 1, and the maximum localization error was 9.00 mm at damage Level 3. Therefore, it could be considered that the method in the paper can achieve accurate location of simulated damages on the structure.

#### 5.2.2. Location Awareness for Impact Damage and Self-Healing States

**Figure 14:**
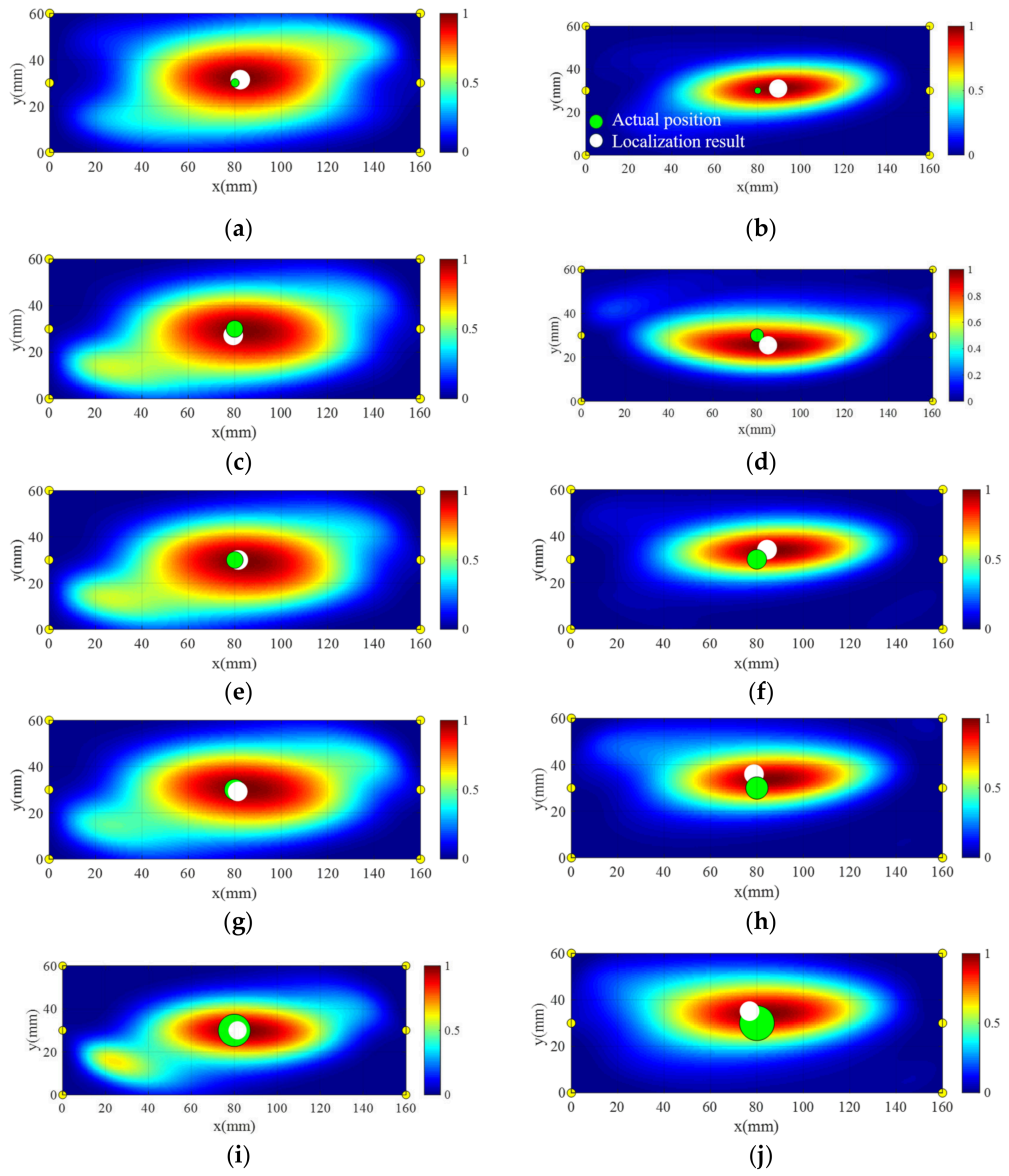
Imaging localization results of impact damage and self-healing regions under different impact energy levels: (**a**) 2 J damaged state; (**b**) 2 J self-healing state; (**c**) 4 J damaged state; (**d**) 4 J self-healing state; (**e**) 6 J damaged state; (**f**) 6 J self-healing state; (**g**) 8 J damaged state; (**h**) 8 J self-healing state; (**i**) 10 J damaged state; and (**j**) 10 J self-healing state.

The imaging location results of the damage position after bearing 2 J impact energy were shown in Figure 14a, and the result of the self-healing position after placing the structure in the healing environment is shown in Figure 14b. According to the error calculation method in the paper, the localization errors of these two states were 2.64 mm and 8.56 mm, respectively. This showed that the imaging location of impact damage location and self-healing location could be achieved according to the method in the article. In order to verify the imaging location results of impact damaged states and self-healing states at different impact energy levels, 4 J, 6 J, 8 J, and 10 J impact loads were applied to the structure, respectively. The actual damage locations and self-healing locations, along with the results of imaging location, are shown in Figure 14c–j.

The localization errors at different impact energy levels and different states were obtained according to the error calculation method proposed in the paper, as shown in Table 1. Among them, the maximum localization error of the damaged state was found to be 2.83 mm for 4 J impact energy, and the minimum error was found to be 1.47 mm for 8 J impact energy. On the other hand, the maximum localization error of self-healing states was found to be 8.56 mm for 2 J impact energy, and the minimum error was found to be 3.78 mm for 6 J impact energy. In all cases, the maximum location error was about 5.35% of the longest distance between sensors, meaning that the position of damage and self-healing could be located when the structure was subjected to impact loading with different energy levels.

## 6. Conclusions

This study developed and validated a multifunctional composite structural framework that integrates thermally activated self-healing capability with piezoelectric GW-based structural health monitoring. The proposed framework enables state awareness of self-healing composite structures by monitoring the evolution from the healthy state to the damaged state and then to the self-healing state. Based on the experimental results, the main conclusions are summarized as follows.

First, simulated damage with different sizes significantly affected the GW responses. As the damaged area increased, the signal amplitude gradually decreased. The A0 mode showed higher sensitivity than the S0 mode, with a maximum amplitude variation of 41.6%. The proposed state index increased with damage size, demonstrating its capability to characterize damage extension. The imaging method achieved a minimum localization error of 5.00 mm for simulated damage. Second, the GW responses under healthy, damaged, and self-healing states showed distinguishable differences. For the 2 J barely visible impact damage case, the maximum amplitude variations relative to the healthy state were 4.5% in the damaged state and 18.4% in the self-healing state. The higher state index in the self-healing state indicates that the healed region was not identical to the original healthy structure, because the polymerized healing agent altered the local material continuity and wave propagation characteristics. Third, the proposed imaging method accurately localized both impact damage and self-healing regions. For the 2 J impact case, the localization errors of the damaged and self-healing states were 2.64 mm and 8.56 mm, respectively. For impact energies from 4 J to 10 J, the maximum localization errors of the damaged and self-healing states were 2.83 mm and 6.73 mm, respectively, corresponding to approximately 1.77% and 4.20% of the maximum sensor distance.

Overall, the proposed method extends conventional GW-based monitoring from binary damage detection to multi-state evaluation of multifunctional self-healing composite structures. Future work will focus on damage-type identification and online monitoring of the self-healing process to further reveal the relationship among damage initiation, healing agent release, polymerization, and guided wave response reconstruction.

## Figures and Tables

**Table 1:** The localization errors at different impact energy and different states.

Impact Energy	Damaged States				Self-Healing States			
	Actual Location (mm)	Identified Location (mm)	*ε_loc_* (mm)	*η_loc_* (%)	Actual Location (mm)	Identified Location (mm)	*ε_loc_* (mm)	*η_loc_* (%)
2 J	(80.00, 30.00)	(82.25, 31.39)	2.64	1.65	(80.00, 30.00)	(88.50, 31.00)	8.56	5.35
4 J	(80.00, 30.00)	(79.38, 27.24)	2.83	1.77	(80.00, 30.00)	(85.00, 25.50)	6.73	4.20
6 J	(80.00, 30.00)	(81.50, 30.00)	1.50	0.94	(80.00, 30.00)	(84.39, 34.17)	6.05	3.78
8 J	(80.00, 30.00)	(81.26, 29.25)	1.47	0.92	(80.00, 30.00)	(78.73, 35.94)	6.07	3.80
10 J	(80.00, 30.00)	(81.50, 30.00)	1.50	0.94	(80.00, 30.00)	(76.81, 35.16)	6.07	3.79

## Data Availability

The original contributions presented in this study are included in the article. Further inquiries can be directed to the corresponding authors.

## References

[1] Ren Y., Tao J., Xue Z. (2020). Design of a large-scale piezoelectric transducer network layer and its reliability verification for space structures. Sensors.

[2] Nie K., Liu Y., Dai Y. (2015). Closed-form solution for the postbuckling behavior of long unsymmetrical rotationally-restrained laminated composite plates under inplane shear. Compos. Struct..

[3] Li N., Chen P.H. (2016). Experimental investigation on edge impact damage and Compression-After-Impact (CAI) behavior of stiffened composite panels. Compos. Struct..

[4] Balasubramaniam K., Sikdar S., Ziaja D., Jurek M., Soman R., Malinowski P. (2023). A global-local damage localization and quantification approach in composite structures using ultrasonic guided waves and active infrared thermography. Smart Mater. Struct..

[5] Ji R., Zhao L., Wang K., Liu F., Gong Y., Zhang J. (2021). Effects of debonding defects on the post-buckling and failure behaviors of composite stiffened panel under uniaxial compression. Compos. Struct..

[6] Deane S., Avdelidis N.P., Ibarra-Castanedo C., Zhang H., Nezhad H.Y., Williamson A.A., Mackley T., Davis M.J., Maldague X., Tsourdos A. (2019). Application of NDT thermographic imaging of aerospace structures. Infrared Phys. Technol..

[7] Wang C., Chen Z., Silberschmidt V.V., Roy A. (2018). Damage accumulation in braided textiles-reinforced composites under repeated impacts: Experimental and numerical studies. Compos. Struct..

[8] Mizukami K., bin Ibrahim A.S., Ogi K., Matvieieva N., Kharabet I., Schulze M., Heuer H. (2019). Enhancement of sensitivity to delamination in eddy current testing of carbon fiber composites by varying probe geometry. Compos. Struct..

[9] Samir K., Brahim B., Capozucca R., Wahab M.A. (2018). Damage detection in CFRP composite beams based on vibration analysis using proper orthogonal decomposition method with radial basis functions and cuckoo search algorithm. Compos. Struct..

[10] Wallentine S.M., Uchic M.D. (2018). A study on ground truth data for impact damaged polymer matrix composites. AIP Conference Proceedings.

[11] Soman R., Radzienski M., Kudela P., Ostachowicz W. (2022). Guided waves-based damage localization based on mode filtering using fiber Bragg grating sensors. Smart Mater. Struct..

[12] Tenreiro A.F.G., Lopes A.M., da Silva L.F. (2022). A review of structural health monitoring of bonded structures using electromechanical impedance spectroscopy. Struct. Health Monit..

[13] Bhuiyan Y., Lin B., Giurgiutiu V. (2018). Acoustic emission sensor effect and waveform evolution during fatigue crack growth in thin metallic plate. J. Intell. Mater. Syst. Struct..

[14] Jing H., Yuan S., Ren Y., Chen J., Wang Y. (2026). A progressive damage model-assisted GW-Gaussian process SHM method for complex composite damage quantification. Compos. Part B Eng..

[15] Yuan S., Meng Y., Chen J., Ren Y., Wu Q. (2025). Digital twin evolution mechanism for individual aircraft life prognosis enhanced by on-line hybrid monitoring. Reliab. Eng. Syst. Saf..

[16] Shao F., Li J., Ouyang L., Ren Y., Qiu L., Yuan S. (2025). Unsupervised adaptive feature enhancement network for reliable damage alarm using guided waves under time-varying conditions. Int. J. Smart Nano Mater..

[17] Xu Y., Chen J., Jing H., Zhao G., Yuan S. (2026). Heteroscedastic guided-wave quantification model for crack monitoring in stepped structures. Int. J. Mech. Sci..

[18] Zhang B., Sun X., Eaton M., Marks R., Clarke A., Featherston C., Kawashita L., Hallett S. (2017). An integrated numerical model for investigating guided waves in impact-damaged composite laminates. Compos. Struct..

[19] Leckey C.A., Wheeler K.R., Hafiychuk V.N., Hafiychuk H., Timuçin D.A. (2018). Simulation of guided-wave ultrasound propagation in composite laminates: Benchmark comparisons of numerical codes and experiment. Ultrasonics.

[20] Zheng K., Li Z., Ma Z., Chen J., Zhou J., Su X. (2019). Damage detection method based on Lamb waves for stiffened composite panels. Compos. Struct..

[21] Gao D., Jiao Y., Wu Z., Zheng Y., Zhang J. (2020). Impedance-and Lamb-Waves-Based Health Condition Monitoring Approach for Composite Cryotanks. J. Spacecr. Rocket..

[22] Chen J., Xu Y., Yuan S., Qin Z. (2023). Guided wave characteristic research and probabilistic crack evaluation in complex multi-layer stringer splice joint structure. Sensors.

[23] Yang X., Xue Z., Zheng H., Qiu L., Xiong K. (2022). Mechanic-electric-thermal directly coupling simulation method of lamb wave under temperature effect. Sensors.

[24] Qing X., Li W., Wang Y., Sun H. (2019). Piezoelectric transducer-based structural health monitoring for aircraft applications. Sensors.

[25] Polverino A., Perfetto D., Caputo F., Zarouchas D., De Luca A. (2026). Damage index selection for ultrasonic guided waves based structural health monitoring system. Procedia Struct. Integr..

[26] Rahul V., Alokita S., Jayakrishna K., Kar V.R., Rajesh M., Thirumalini S., Manikandan M. (2019). Structural health monitoring of aerospace composites. Structural Health Monitoring of Biocomposites, Fibre-Reinforced Composites and Hybrid Composites.

[27] Moll J., De Marchi L., Kexel C., Marzani A. (2017). High resolution defect imaging in guided waves inspections by dispersion compensation and nonlinear data fusion. Acta Acust. United Acust..

[28] Zheng F., Chen J., Yuan S., Xu Q., Qiu L. (2024). Guided wave-MUSIC based damage monitoring method for complex composite structures. Int. J. Mech. Sci..

[29] Gao D., Ma Y., Wu Z., Zheng Y., Lu H. (2021). Guided Wave Based Damage Detection Method for Aircraft Composite Structures under Varying Temperatures. Struct. Durab. Health Monit..

[30] Wang Q., Xu Y., Su Z., Cao M., Yue D. (2019). An enhanced time-reversal imaging algorithm-driven sparse linear array for progressive and quantitative monitoring of cracks. IEEE Trans. Instrum. Meas..

[31] Zheng F., Yuan S. (2024). Guided lamb wave array time-delay-based MUSIC algorithm for impact imaging. Sensors.

[32] Qiu L., Liu M., Qing X. (2013). A quantitative multi-damage monitoring method for large-scale complex composite. Struct. Health Monit..

[33] Xu C., Yang Z., Tian S., Chen X. (2019). Lamb wave inspection for composite laminates using a combined method of sparse reconstruction and delay-and-sum. Compos. Struct..

[34] Yue N., Khodaei Z.S., Aliabadi M.H. (2021). Damage detection in large composite stiffened panels based on a novel SHM building block philosophy. Smart Mater. Struct..

[35] Kwak B.S., Lee G.E., Kang G.S., Kweon J.H. (2019). An investigation of repair methods for delaminated composite laminate under flexural load. Compos. Struct..

[36] Zhang C., Liu R., Tao C., Zhang C., Ji H., Qiu J. (2022). Design, construction, and modeling of aircraft door sealing plate based on SMAs. Int. J. Smart Nano Mater..

[37] Yang Y., Wang Y., Yao T., Feng X. (2022). A flexible and smart shape memory alloy composite sheet based on efficient and bidirectional thermal management. Int. J. Smart Nano Mater..

[38] White S.R., Sottos N.R., Geubelle P.H., Moore J.S., Kessler M.R., Sriram S.R., Brown E.N., Viswanathan S. (2001). Autonomic healing of polymer composites. Nature.

[39] Kessler M.R., White S.R. (2001). Self-activated healing of delamination damage in woven composites. Compos. Part A Appl. Sci. Manuf..

[40] Yin T., Rong M.Z., Wu J., Chen H., Zhang M.Q. (2008). Healing of impact damage in woven glass fabric reinforced epoxy composites. Compos. Part A Appl. Sci. Manuf..

[41] Patel A.J., Sottos N.R., Wetzel E.D., White S.R. (2010). Autonomic healing of low-velocity impact damage in fiber-reinforced composites. Compos. Part A Appl. Sci. Manuf..

[42] Qiu L., Yuan S. (2009). On development of a multi-channel PZT array scanning system and its evaluating application on UAV wing box. Sens. Actuators A Phys..

[43] Su Z., Ye L., Lu Y. (2006). Guided Lamb waves for identification of damage in composite structures: A review. J. Sound Vib..

